# Translating Research into Reality: Elimination of Lymphatic Filariasis from Haiti

**DOI:** 10.4269/ajtmh.16-0669

**Published:** 2017-10-18

**Authors:** Patrick J. Lammie, Mark L. Eberhard, David G. Addiss, Kimberly Y. Won, Madsen Beau de Rochars, Abdel N. Direny, Marie Denise Milord, Jack Guy Lafontant, Thomas G. Streit

**Affiliations:** 1 Centers for Disease Control and Prevention, Atlanta, Georgia;; 2 Hospital Ste. Croix, Leogane, Haiti;; 3 Ministry of Health and Population, Haiti;; 4 University of Notre Dame, Notre Dame, Indiana

## Abstract

Research provides the essential foundation of disease elimination programs, including the global program to eliminate lymphatic filariasis (GPELF). The development and validation of new diagnostic tools and intervention strategies, critical steps in the evolution of GPELF, required a global effort. Lymphatic filariasis research in Haiti involved many partners and was directly linked to the development of the national elimination program and to the success achieved to date. Ongoing research efforts involving many partners will continue to be important in resolving the challenges faced by the program today in its final efforts to achieve elimination.

## INTRODUCTION

Lymphatic filariasis (LF) is the target of a global elimination program, aimed at preventing transmission of a disease which in its chronic phases, causes incapacitating lymphedema, elephantiasis, and hydrocele. After more than 15 years of concerted effort, Haiti is poised to eliminate LF. The country has successfully scaled up mass drug administration (MDA) to achieve 100% geographic coverage and is now carrying out World Health Organization (WHO)-recommended transmission assessment survey (TAS) to stop MDA across many areas of the country^1,2^ This success reflects not only the strong commitment over many years by resilient partners, but also the robust foundation of a program built on decades of investment in field and laboratory research. Research is at the core of all successful disease elimination programs, including the Global Program to Eliminate LF (GPELF) through the development and validation of effective intervention tools and strategies. For GPELF, key advances including the development of new treatment strategies based on single-dose treatment and rapid diagnostic tools, were made with Haiti serving as a key partner.^3^ Furthermore, research done in Haiti provided the genesis of the public health effort to eliminate LF in the country. Engagement by the U.S. Centers for Disease Control and Prevention (CDC) on LF in Haiti, with the support of the University of Notre Dame and Ministry of Health, provides an excellent case study of how research activities can support the development and evolution of disease elimination programs. This review will describe how the research, carried out in the context of a global research initiative, contributed to the development of Haiti’s program to eliminate LF.

## INITIAL RESEARCH EFFORTS

The genesis of CDC’s research effort in Haiti was National Institutes of Health (NIH) funding to Tulane University for an International Center for Medical Research (ICMR), which included funding for research activities in Colombia and Haiti in the 1980s. CDC staff members were involved in this research and on the closure of the ICMR, sustained the research with funding from CDC, NIH, WHO’s Special Program for Research and Training in Tropical Diseases (TDR), and other donors. Early work focused on studies of the epidemiology of LF and led to key observations, including demonstration that 1) individuals with microfilaremia as low as 1/mL could still infect mosquitoes, 2) a small proportion of individuals continued to be microfilaremic even after repeated 12-day courses of diethylcarbamazine (DEC), and 3) children born to microfilaremic mothers were more likely to acquire infection than children born to amicrofilaremic mothers.^4–9^ These studies established a field presence and framework to investigate public health strategies to control LF.

## TREATMENT STRATEGY

Historically, treatment of LF was based on a 12-day course of DEC (6 mg/kg). Although a number of highly successful mass treatment campaigns were carried out with DEC, these were typically based on modified dosing strategies and were relatively limited in scope.^10^ Where community residents were provided with the full 12-day complement of tablets, treatment compliance was generally low and where treatment was directly observed, the number of persons who could be followed up effectively by a single team was quite limited. Alternative treatment strategies, including spaced weekly doses were tested in Haiti and found to be superior to daily dosing, but this approach afforded no advantages in terms of the logistics of treating on a large scale.^11^ The inability to practically deliver 12 days of therapy based on directly observed treatment as well as the demonstration of the effectiveness of single-dose ivermectin for the treatment of onchocerciasis, another filarial infection, stimulated a series of multicountry studies to investigate the safety and efficacy of single-dose treatments, compared with a standard 12-day course of DEC. WHO-TDR and Merck and Co. were major supporters of these studies and different dosing strategies were used to determine if ivermectin had an adulticidal effect and to define the optimal microfilaricidal dose.^12–15^ An unexpected outcome of these studies was the demonstration that a single dose of DEC was as effective as a 12-day course. These studies provided the critical evidence to support the concept that annual mass treatment based on single-dose therapy represented a practical intervention strategy for the control of LF and raised new questions about the potential for coadministered combinations of drugs. CDC provided funding for studies in Haiti to investigate the potential contribution of albendazole (ALB) to antifilarial drug combinations based on evidence from pilot studies that multiple doses had a pronounced adulticidal effect.^16^

Recognizing the broad spectrum deworming benefits of ALB, randomized controlled trials of ivermectin plus ALB as well as DEC plus ALB combinations in Haiti were carried out in school-aged children to monitor the nutritional impact of treatment as well as the effect on soil transmitted helminths (STH). These studies documented the effectiveness of regimens including ALB for the treatment of both LF and STH, and in conjunction with studies carried out in other settings, established the framework of the MDA strategy for GPELF.^17–19^ In this context, it is also important to identify GPELF as the first global program to represent an integrated neglected tropical disease (NTD) program, through its impact on both LF and STH, a step that anticipated the later emphasis on NTDs as diseases of poverty that could be addressed by preventive chemotherapy.^20–22^

## DIAGNOSTICS STUDIES

A key requirement for conducting mass treatment is defining the populations in need of treatment. Diagnosis of LF, traditionally, required the demonstration of microfilariae. In Haiti, and in much of the world where *Wuchereria bancrofti* is transmitted, microfilaremia is nocturnal, necessitating night blood surveys to diagnose infection. These surveys represent an inconvenience to both the survey teams and the populations being surveyed and were not well accepted by many communities. The discovery that monoclonal antibodies developed against nonhuman filarial parasites recognized a circulating *W. bancrofti* antigen that could be detected in blood collected at any time of the day led to important insights in the epidemiology of filarial infection.^23,24^ In Haiti, antigen assays were incorporated into field work and clinical trials as soon as tests became available. These studies documented that 1) infection prevalence as measured by antigenemia was much higher than microfilaremia; 2) children as young as 2 years of age were acquiring infection; 3) chemotherapy led to slow and partial reductions in antigenemia; and 4) most lymphedema and elephantiasis patients were antigen-negative and thus, would not be expected to benefit from antifilarial chemotherapy.^25–30^ A revolutionary advance for LF programs was the introduction of a rapid diagnostic test, the immunochromatographic card test (ICT), which opened the door to daytime surveys to map the distribution of LF across all countries where LF was known or suspected.^31^ In Haiti, antigen surveys were conducted in schools across the country and documented that LF was more widespread than anticipated and follow-up studies documented transmission in several of these low prevalence settings.^32,33^ The widespread distribution of LF and LF transmission in Haiti led the Ministry of Health and Population (MSPP) to conclude that a program to eliminate LF would have to be national in scope.

## MASS DRUG ADMINISTRATION

The recognition that single-dose treatment was efficacious opened the door to the development and testing of community-based mass treatment campaigns. CDC supported operational research to evaluate MDA approaches, initially based on house-to-house distribution of ivermectin in the community of Belloc near Leogane. The house-to-house strategy proved challenging due to the number of household visits required to achieve acceptable coverage. As a result, MDA strategies employing active social mobilization campaigns and the use of centrally located distribution posts were tested and adopted as the foundation of the CDC-funded Leogane Demonstration Project, a comprehensive study designed to determine if five annual rounds of MDA would be sufficient to eliminate LF transmission in the commune.

When research studies demonstrated the effectiveness of drug combinations that included ALB, WHO asked that countries generate safety data from carefully monitored populations following administration of either ivermectin or DEC plus ALB. Combinations that included ivermectin were used in sub-Saharan Africa where onchocerciasis was endemic; in Haiti and the rest of the world where only LF was endemic, DEC was used. Safety studies in Leogane documented that the DEC + ALB combination had an acceptable safety profile.^34^ Adverse events included fever, headache, and malaise associated with the killing of microfilariae as well as localized reactions associated with the killing of adult worms. Based on similar findings from other countries, WHO gave a “green light” to the use of MDA strategies based on the two drug combinations.^35^ In Haiti, MSPP expressed concern about the treatment of women of child bearing age with ALB, since ALB is not recommended during the first trimester, when pregnancy may not be recognized. Consequently, only men and children received both drugs for the first 2 years of MDA in Leogane (MDAs in 2000 and 2001). Women were treated with DEC alone. As part of the monitoring and evaluation (M and E) strategy developed for the Leogane project, stool samples were collected to monitor the impact of MDA on STH infections. These data documented the differential benefit of MDA for men and children who received ALB, compared with women who did not and led MSPP to reverse their earlier decision and make women eligible to receive ALB, both in Leogane and other communes as the program scaled up.^36^ When funds from the Bill & Melinda Gates Foundation to the University of Notre Dame made it possible to begin to expand MDA to other high-prevalence communes, women in these settings were eligible to receive the dual drug combination. As documented in other publications, scaling up MDA to reach all LF-endemic communes was hampered by limitations in funding, civil strife, and natural disasters.^1^ Full national coverage was not achieved until after the catastrophic 2010 earthquake. Postearthquake funding from CDC and other donors supported the scale-up of MDA in Port au Prince, a major logistic effort and the last piece needed to achieve national coverage.^37^

## MONITORING AND EVALUATION

The Leogane Demonstration project included a rigorous M and E component to inform requirements for the scale-up of the national program. Adverse events, coverage, and cost were monitored for each round of MDA.^38–41^ Surveys of microfilaremia, antigenemia, and STH burden were conducted in sentinel sites on an annual basis and filarial infection in mosquitoes was monitored periodically. These surveys documented the impact of MDA on STH as well as filarial infection in humans and mosquitoes.^36,39,42^ After five rounds of MDA, microfilaria prevalence was less than 1%^43^; however, civil strife led to interruptions in funding and MDA, not only for Leogane, but for other high prevalence or “zone rouge” communes as well. Follow-up surveys in 2007 demonstrated a significant recrudescence of microfilaremia and antigenemia in Leogane sentinel sites, leading to the conclusion that a single round of missed MDA set the program back by at least 2 years.^44^

Even with the setbacks resulting from the interrupted MDA, there was a growing recognition that 5 years of MDA might not be sufficient to interrupt transmission in the “zone rouge” communes. Data from coverage surveys and the evidence of persistent transmission in Leogane also led to concerns that systematic noncompliance could be contributing to persistent transmission by maintaining a reservoir of infection.^40,45,46^ Additional surveys demonstrated that noncompliance rates differed across communities and were associated with greater prevalence of antigenemia.^47^ Systematic noncompliance was driven by a complex array of factors, including fear of adverse reactions to drug treatment and mistrust of institutions. Although these challenges are unlikely to be unique to Leogane, it is possible that the initial restriction of ALB use to children and men in the first 2 years of the program exacerbated the problem in Leogane. These experiences highlighted the need for all programs to develop effective communication strategies and clear messages to support MDA programs.

After years of challenges with scaling up MDA, the LF program in Haiti achieved full geographic coverage in 2012. After multiple rounds of treatment of at risk populations, national LF elimination programs must be able to assess whether MDA has succeeded in lowering the prevalence of infection to a level where transmission is likely no longer sustainable. Determining whether MDA can be stopped requires the use of appropriate diagnostic tools and robust survey methodologies. Research activities conducted in *W. bancrofti* endemic countries, including Haiti, led to the selection of the ICT as the diagnostic tool to assess whether MDA can safely be stopped.^48^ Subsequently, the TAS was designed and is currently the WHO recommended survey for making the programmatic decision to stop or continue MDA.^49^ In Haiti many areas of the country are meeting the criteria for conducting TAS. In addition to providing important information for the LF program, the TAS platform provides excellent opportunities to collect additional public health data. Haiti is exploiting this opportunity by piloting an integrated TAS that includes malaria and STH assessments to guide public health decisions for these diseases.^50^

## LYMPHEDEMA MANAGEMENT

In early stages of the CDC research activities in Leogane, patients with lymphedema and elephantiasis were frequently included in house-to-house and clinic-based night blood surveys for microfilaremia. Though such patients were almost universally microfilaria-negative, they were typically offered a standard 12-day course of DEC in the hopes that they would derive some benefit from antifilarial treatment. That these patients never benefitted clinically was a puzzle until the introduction of antigen testing, as noted earlier, when it became clear that 95% of lymphedema patients in Haiti were antigen-negative and thus, had no evidence of active LF.^26,28,51^ This led to the realization that lymphedema patients would not benefit from MDA and raised questions about how to provide appropriate care for these patients. At this same time, pioneering clinical work by Gerusa Dreyer in Brazil demonstrated that recurrent skin infections were responsible for acute attacks of adenolymphangitis (ADL) in lymphedema patients and for disease progression.^52–55^ This recognition led to the development of strategies to manage lymphedema based on prevention of ADL through improved skin hygiene.^56–58^ Collaboration with Dreyer introduced these principles of self-care to a newly developed lymphedema clinic in Leogane and follow-up of these patients also documented a reduced frequency of ADL as well as decreased skin pathology and inflammation.^59–61^ As a result of these and other studies, GPELF was based on two pillars—one focused on MDA and the second on providing access to appropriate care for patients already affected by filarial disease.^62^

## LOOKING FORWARD: OPERATIONAL RESEARCH NEEDS FOR THE LAST MILE

Though the LF program has matured, both in Haiti and in many countries around the world, the need for operational research has not ended. Persistent transmission, even after 10 years of MDA in several of the “zone rouge” communes, represents a particular challenge for Haiti. DEC-fortified salt, an exceptionally effective intervention in early pilot studies in Haiti,^63^ is still being investigated as one potential solution. The recent demonstration of the increased efficacy of triple drug therapy (ivermectin plus ALB plus DEC) raises hopes that this drug combination may represent another option to solve the problem of persistent transmission.^64^ Trials to demonstrate the safety, community acceptability, and efficacy of this combination are now planned for Haiti and other countries. Even if this strategy proves to be successful, stopping MDA does not represent the end of the LF program; post-MDA surveillance is needed to demonstrate that LF does not return. Current surveillance efforts are based on repeated TAS, but these surveys are not powered to detect changes in antigen prevalence. New diagnostic tools and surveillance platforms continue to be needed and Haiti is a logical place to test and validate these new approaches.^65,66^ The new focus on malaria elimination and the requirement for enhanced surveillance to achieve this target may provide Haiti with new opportunities to test options for integrated surveillance that will help to achieve both LF and malaria elimination. If focal MDA emerges as an effective strategy to eliminate malaria, the long-standing experience with LF MDA will also surely be advantageous. LF elimination, though not fully complete, is no longer a distant goal, but is approaching rapidly. The success of this program reflects the commitments of partners and communities over many years and through many challenges, but also represents the dividends from investments in research over this same period of time. Finally, it is important to note that the Haitian population is not alone realizing the benefits of the research that they have so patiently supported over the years; many LF-affected communities around the world are benefitting as well.

## References

[1] Oscar R, 2014 Haiti national program for the elimination of lymphatic filariasis: a model of success in the face of adversity. PLoS Negl Trop Dis 8: e2915. 25032697 10.1371/journal.pntd.0002915 PMC4102456

[2] Lemoine JF, 2016. Controlling Neglected Tropical Diseases (NTDs) in Haiti: implementation strategies and evidence of their success. PLoS Negl Trop Dis 10*:* e0004954. 10.1371/journal.pntd.0004954 PMC5051938 27706162

[3] Ichimori K King J Engels D Yajima A Mikhailov A Lammie P Ottesen E, 2014 Global programme to eliminate lymphatic filariasis: the processes underlying programme success. PLoS Negl Trop Dis 8: e3328. 25502758 10.1371/journal.pntd.0003328 PMC4263400

[4] Lowrie RC Eberhard ML Lammie PJ Raccurt CP Katz SP Duverseau YT, 1989 Uptake and development of *Wuchereria bancrofti* in *Culex quinquefasciatus* that fed on Haitian carriers with different microfilaria densities. Am J Trop Med Hyg 41: 429–435. 2679171 10.4269/ajtmh.1989.41.429

[5] Eberhard ML Lowrie RC Lammie PJ, 1988 Persistence of microfilariae in *Wuchereria bancrofti* carriers following DEC-C treatment. Trop Med Parasitol 39: 128–130.3051291

[6] Eberhard ML Lammie PJ Dickinson CM Roberts JM, 1991 Evidence of nonsusceptibility to diethylcarbamazine in *Wuchereria bancrofti*. J Infect Dis 163: 1157–1160. 2019765 10.1093/infdis/163.5.1157

[7] Eberhard ML Dickerson JW Hightower AW Lammie PJ, 1991 Bancroftian filariasis: long term effects of treatment with diethylcarbamazine (DEC) in a Haitian population. Am J Trop Med Hyg 45: 728–733. 1763800 10.4269/ajtmh.1991.45.728

[8] Lammie PJ Hitch WL Walker EM Hightower AW Eberhard ML, 1991 Maternal infection as a risk factor for infection in offspring. Lancet 337: 1005–1006. 1673168 10.1016/0140-6736(91)92661-k

[9] Hightower AW Lammie PJ Eberhard ML, 1993 Maternal filarial infection—a persistent risk factor for infection in offspring? Parasitol Today 9: 418–421. 15463683 10.1016/0169-4758(93)90051-g

[10] Sasa M, 1976 *Human Filariasis*. Baltimore, MD: University Park Press.

[11] Eberhard ML Lammie PJ Roberts JM Lowrie RC, 1989 Effectiveness of spaced doses of diethylcarbamazine citrate in the control of bancroftian filariasis. Trop Med Parasitol 40: 111–113. 2672282

[12] Richards FO Eberhard ML Bryan R McNeeley DF Lammie PJ McNeeley MB Bernard Y Hightower AW Spencer HC, 1991 Comparison of ivermectin and diethylcarbamazine for adulticidal activity in Haitian bancroftian filariasis. Am J Trop Med Hyg 44: 3–10. 1996738 10.4269/ajtmh.1991.44.3

[13] Addiss DG Eberhard ML Lammie PJ Hitch WL Spencer HC, 1991 Tolerance of single high-dose ivermectin for treatment of lymphatic filariasis. Trans R Soc Trop Med Hyg 85: 256–257. 10.1016/0035-9203(91)90049-5 1887489

[14] Eberhard ML Hightower AW McNeeley DF Lammie J, 1992 Long-term suppression of microfilaremia following ivermectin treatment. Trans Roy Soc Trop Med 86: 287–288. 10.1016/0035-9203(92)90312-z 1412655

[15] Addiss DG Eberhard ML Lammie PJ McNeeley MB Lee SH McNeeley DM Spencer HC, 1993 Comparative trial of clearing-dose and single high-dose ivermectin and diethylcarbamazine for treatment of lymphatic filariasis. Am J Trop Med Hyg 48: 178–185. 8447520 10.4269/ajtmh.1993.48.178

[16] Jayakody RL De Silva CS Weerasinghe WM, 1993 Treatment of bancroftian filariasis with albendazole: evaluation of efficacy and adverse reactions. Trop Biomed 10: 19–24.

[17] Addiss DG Beach MJ Streit TG Lutwick S LeConte FH Lafontant JG Lammie PJ, 1997 Randomised placebo-controlled comparison of ivermectin and albendazole alone and in combination for *Wuchereria bancrofti* microfilaraemia in Haitian children. Lancet 350: 480–484. 9274584 10.1016/S0140-6736(97)02231-9

[18] Beach MJ Streit TG Addiss DG Prospere R Roberts JM Lammie PJ, 1999 Assessment of combined ivermectin and albendazole for treatment of intestinal helminth and *Wuchereria bancrofti* infections in Haitian school children. Am J Trop Med Hyg 60: 479–486. 10466981 10.4269/ajtmh.1999.60.479

[19] Fox LM Furness BW Hase JK Dardith D Brissau J-M Milord MD Lafontant JG Addiss DG Lammie P Beach MJ, 2005 Tolerance and efficacy of combined diethylcarbamazine and albendazole for treatment of *Wuchereria bancrofti* and intestinal helminth infections in Haitian children. Am J Trop Med Hyg 73*:* 115–121. 16014845

[20] Molyneux DH Hotez PJ Fenwick A, 2005 “Rapid-impact interventions”: how a policy of integrated control for Africa’s neglected tropical diseases could benefit the poor. PLoS Med 11: e336. 10.1371/journal.pmed.0020336 PMC1253619 16212468

[21] Hotez PJ Molyneux DH Fenwick A Kumaresan J Sachs SE Sachs JD Savioli L, 2007 Control of neglected tropical diseases. N Engl J Med 357: 1018–1027 [Review]. 17804846 10.1056/NEJMra064142

[22] Hotez PJ Fenwick A Savioli L Molyneux DH, 2009 Rescuing the bottom billion through control of neglected tropical diseases. Lancet 373: 1570–1575 [Review]. 19410718 10.1016/S0140-6736(09)60233-6

[23] More SJ Copeman DB, 1990 A highly specific and sensitive monoclonal antibody-based ELISA for the detection of circulating antigen in bancroftian filariasis. Trop Med Parasitol 41: 403–406. 2075384

[24] Ramzy RM Gad AM Faris R Weil GJ, 1991 Evaluation of a monoclonal-antibody based antigen assay for diagnosis of *Wuchereria bancrofti* infection in Egypt. Am J Trop Med Hyg 44: 691–695. 1858970 10.4269/ajtmh.1991.44.691

[25] Weil GJ Lammie PJ Richards FO Jr Eberhard ML, 1991 Changes in circulating parasite antigen levels after diethylcarbamazine treatment of bancroftian filariasis. J Infect Dis 164: 814–816. 1894943 10.1093/infdis/164.4.814

[26] Lammie PJ Addiss DG Leonard G Hightower AW Eberhard ML, 1993 Heterogeneity in filarial specific immune responsiveness among patients with lymphatic obstruction. J Infect Dis 167: 1178–1183. 8486952 10.1093/infdis/167.5.1178

[27] Lammie PJ Hightower AW Eberhard ML, 1994 The age-specific prevalence of antigenemia in a *Wuchereria bancrofti*-exposed population. Am J Trop Med Hyg 51: 348–355. 7943556 10.4269/ajtmh.1994.51.348

[28] Addiss DG Dimock KA Eberhard ML Lammie PJ, 1995 Clinical, parasitologic and immunologic observations of patients with hydrocele and elephantiasis in an area with endemic lymphatic filariasis. J Infect Dis 171: 755–758. 7876636 10.1093/infdis/171.3.755

[29] Eberhard ML Hightower AW Addiss DG Lammie PJ, 1997 Clearance of *Wuchereria bancrofti* antigen after treatment with diethylcarbamazine or ivermectin. Am J Trop Med Hyg 57: 483–486. 9347968 10.4269/ajtmh.1997.57.483

[30] Lammie PJ Reiss MD Dimock KA Streit TG Roberts JM Eberhard ML, 1998 Longitudinal analysis of the development of filarial infection and antifilarial immunity in a cohort of Haitian children. Am J Trop Med Hyg 59: 217–221. 9715935 10.4269/ajtmh.1998.59.217

[31] Weil GJ Lammie PJ Weiss N, 1997 The ICT filariasis test: a rapid format antigen test for diagnosis of bancroftian filariasis. Parasitol Today 13: 401–404. 15275155 10.1016/s0169-4758(97)01130-7

[32] Beau de Rochars MV Milord MD St Jean Y Désormeaux AM Dorvil JJ Lafontant JG Addiss DG Streit TG, 2004 Geographic distribution of lymphatic filariasis in Haiti. Am J Trop Med Hyg 71: 598–601. 15569791

[33] Drexler N Washington C Lovegrove M Grady C Milord MD Streit T Lammie P, 2012 Secondary mapping of lymphatic filariasis in Haiti: definition of transmission foci in low prevalence settings. PLoS Negl Trop Dis 6: e1807. 23071849 10.1371/journal.pntd.0001807 PMC3469481

[34] McLaughlin SI Radday J Michel MC Addiss DG Beach MJ Lammie PJ Lammie J Rheingans R Lafontant J, 2003 Frequency, severity and costs of adverse reactions following mass treatment for lymphatic filariasis using diethylcarbamazine and albendazole, Leogane, Haiti, 2000. Am J Trop Med Hyg 68: 568–573. 12812348 10.4269/ajtmh.2003.68.568

[35] Horton J, 2000 An analysis of the safety of the single dose, two drug regimens used in programmes to eliminate lymphatic filariasis. Parasitology 121: S147–S160. 11386686 10.1017/s0031182000007423

[36] Beau de Rochars M Direny AD Roberts JM Addiss DG Radday J Beach MJ Streit TG Dardith D Lafontant J Lammie PJ, 2004 Community-wide reduction in prevalence and intensity of intestinal helminths as a collateral benefit of lymphatic filariasis elimination programs. Am J Trop Med Hyg 71: 466–470. 15516644

[37] Beau de Rochars MVE Streit TG Desir L Oscar R Lemoine JF Keller A Lammie PJ Purcell N Deming MS 2013 Mass drug administration for the elimination of lymphatic filariasis: Port-au-Prince, Haiti, 2011–2012. MMWR Morb Mortal Wkly Rep 62: 466–468. 23760187 PMC4604845

[38] Mathieu E Deming M Lammie P McLaughlin S Beach M Deodat DJ Addiss D, 2003 Comparison of methods for estimating drug coverage for filariasis elimination, Leogane, Haiti. Trans R Soc Trop Med Hyg 97: 501–505. 15307410 10.1016/s0035-9203(03)80006-8

[39] Beau de Rochars M, 2005 The Leogane, Haiti demonstration project: decreased microfilaremia and program costs after three years of mass drug administration. Am J Trop Med Hyg 73: 888–894. 16282299

[40] Mathieu E Direny A Streit T Beau de Rochars M Addiss D Lammie P, 2006 Trends in participation in three consecutive mass drug administrations in Leogane, Haiti. Trop Med Int Health 11: 862–868. 16772008 10.1111/j.1365-3156.2006.01626.x

[41] Hochberg N Michel MC Lammie PJ Mathieu E Direny AN Beau de Rochars M Addiss DG, 2006 Symptoms reported after mass drug administration for lymphatic filariasis in Leogane, Haiti: lack of association with filarial infection. Am J Trop Med Hyg 75: 928–932. 17123989

[42] Goodman DS Orelus JN Roberts JM Lammie PJ Streit TG, 2003 PCR and mosquito dissection as tools to monitor filarial infection levels following mass treatment. Filaria J 2: 11. 12890288 10.1186/1475-2883-2-11 PMC169178

[43] Grady CA, 2007 Endpoints for lymphatic filariasis programs. Emerg Infect Dis 13: 608–610. 17553278 10.3201/eid1304.061063 PMC2725965

[44] Won KY Beau de Rochars M Kyelem D Streit TG Lammie PJ, 2009 Assessing the impact of a missed MDA in Haiti. PLoS Negl Trop Dis 3: e443. 19707279 10.1371/journal.pntd.0000443 PMC2724684

[45] Mathieu E Lammie PJ Radday J Montilus W Beach MJ Streit T Wendt J Addiss DG, 2004 What influenced people to participate in a mass treatment campaign against lymphatic filariasis? Ann Trop Med Parasitol 98: 703–714. 15509424 10.1179/000349804X3135

[46] Talbot JT Viall A Direny AN Beau de Rochars MVE Addiss DG Streit T Mathieu E Lammie PJ, 2008 Predictors of compliance in mass drug administration for the prevention and treatment of lymphatic filariasis in Leogane, Haiti. Am J Trop Med Hyg 78: 283–288. 18256430

[47] Boyd A, 2010 Factors associated with continuing transmission of lymphatic filariasis in Leogane, Haiti. PLoS Negl Trop Dis 4: e640. 20351776 10.1371/journal.pntd.0000640 PMC2843627

[48] Gass K, 2012 A multicenter evaluation of diagnostic tools to define endpoints for programs to eliminate Bancroftian filariasis. PLoS Negl Trop Dis 6: e1479. 22272369 10.1371/journal.pntd.0001479 PMC3260316

[49] World Health Organization, 2011. *Monitoring and Epidemiological Assessment of Mass Drug Administration in the Global Programme to Eliminate Lymphatic Filariasis: A Manual for National Elimination Programmes*. Geneva, Switzerland: WHO.

[50] Knipes A, 2017 Partnering for impact: integrated transmission assessment surveys for lymphatic filariasis, soil transmitted helminths and malaria in Haiti. PLoS Negl Trop Dis 11: e0005387. 28207792 10.1371/journal.pntd.0005387 PMC5332101

[51] Baird JB Lammie PJ Louis Charles J Streit TG Addiss DG, 2002 Reactivity to bacterial, fungal, and parasite antigens in patients with lymphedema and elephantiasis. Am J Trop Med Hyg 66: 163–169. 12135288 10.4269/ajtmh.2002.66.163

[52] Dreyer G Medeiros Z Netto MJ Leal NC de Castro LG Piessens WF, 1999 Acute attacks in the extremities of persons living in an area endemic for bancroftian filariasis: differentiation of two syndromes. Trans R Soc Trop Med Hyg 93: 413–417. 10674092 10.1016/s0035-9203(99)90140-2

[53] Fox LM Wilson SF Addiss DG Louis-Charles J Beau de Rochars MV Lammie PJ, 2005 Clinical correlates of filarial infection in Haitian children: an association with interdigital lesions. Am J Trop Med Hyg 73: 759–765. 16222022

[54] Dreyer G Addiss D Gadelha P Lapa E Williamson J Dreyer A, 2006 Interdigital skin lesions of the lower limbs among patients with lymphoedema in an area endemic for bancroftian filariasis. Trop Med Int Health 11: 1475–1481. 16930270 10.1111/j.1365-3156.2006.01687.x

[55] Addiss DG Eberhard ML Lammie PJ, 1994 “Filarial” adenolymphangitis without filarial infection. Lancet 343: 597. 10.1016/s0140-6736(94)91547-4 7906341

[56] Dreyer G Norões J Figueredo-Silva J, 2000 New insights into the natural history and pathology of bancroftian filariasis: implications for clinical management and filariasis control programmes. Trans R Soc Trop Med Hyg 94: 594–596. 11198637 10.1016/s0035-9203(00)90200-1

[57] Dreyer G Addiss DG Dreyer P Noroes J, 2002 *Basic Lymphedema Management. Treatment and Prevention of Problems Associated with Lymphatic Filariasis*. Hollis, NH: Hollis Publishing Company.

[58] Dreyer G Norões J Mattos D, 2006 Hope clubs as adjunct therapeutic measure in bancroftian filariasis endemic areas. Rev Soc Bras Med Trop 39: 365–369. 17119752 10.1590/s0037-86822006000400009

[59] Wilson SF Guarner J Valme AL Louis-Charles J Jones TL Addiss DG, 2004 Histopathologic improvement with lymphedema management, Léogâne, Haiti. Emerg Infect Dis 10: 1938–1946. 15550203 10.3201/eid1011.040548 PMC3329004

[60] Addiss DG Louis-Charles J Roberts J LeConte F Wendt JM Milord MD Lammie PJ Dreyer G, 2010 Feasibility and effectiveness of basic lymphedema management in Leogane, Haiti, an area endemic for Bancroftian filariasis. PLoS Negl Trop Dis 4: e668. 20422031 10.1371/journal.pntd.0000668 PMC2857874

[61] Addiss DG Michel MC Michelus A Radday J Billhimer W Louis-Charles J Roberts JM Kramp K Dahl BA Keswick B, 2011 Evaluation of antibacterial soap in the management of lymphoedema in Leogane, Haiti. Trans R Soc Trop Med Hyg 105: 58–60. 20850849 10.1016/j.trstmh.2010.08.011

[62] Seim AR Dreyer G Addiss DG, 1999 Controlling morbidity and interrupting transmission: twin pillars of lymphatic filariasis elimination. Rev Soc Bras Med Trop 32: 325–328. 10380574 10.1590/s0037-86821999000300022

[63] Freeman AR Lammie PJ Houston R Jooste PL Lapointe MD Streit TG Brissau JM Lafontant J Addiss DG, 2001 A community-based trial for the control of lymphatic filariasis and iodine deficiency using salt fortified with diethylcarbamazine and iodine. Am J Trop Med Hyg 65: 865–871. 11791989 10.4269/ajtmh.2001.65.865

[64] Thomsen EK, 2016 Efficacy, safety, and pharmacokinetics of coadministered diethylcarbamazine, albendazole, and ivermectin for treatment of Bancroftian filariasis. Clin Infect Dis 62: 334–341. 26486704 10.1093/cid/civ882

[65] Hamlin KL Moss DM Priest JW Roberts J Kubofcik J Gass K Streit TG Nutman TB Eberhard ML Lammie PJ, 2012 Longitudinal monitoring of the development of antifilarial antibodies and acquisition of *Wuchereria bancrofti* in a highly endemic area of Haiti. PLoS Negl Trop Dis 6: e1941. 23236534 10.1371/journal.pntd.0001941 PMC3516578

[66] Lammie PJ Moss DM Goodhew EB Hamlin K Krolewiecki A West SK Priest JW, 2012 Development of a new diagnostic platform for NTD surveillance. Int J Parasitol 42: 797–800. 22846784 10.1016/j.ijpara.2012.07.002

